# PSMA Receptor-Based PET-CT: The Basics and Current Status in Clinical and Research Applications

**DOI:** 10.3390/diagnostics13010158

**Published:** 2023-01-03

**Authors:** Aadil Adnan, Sandip Basu

**Affiliations:** 1 Radiation Medicine Centre, Bhabha Atomic Research Centre, Tata Memorial Centre Annexe, Jerbai Wadia Road, Parel, Mumbai 400012, India; 2 Homi Bhabha National Institute, Mumbai 400085, India

**Keywords:** ^68^Ga-PSMA, FDG, PET-CT, ^177^Lu-PSMA-617, prostate cancer, metastatic castration-resistant prostate carcinoma, peptide receptor radioligand therapy

## Abstract

Prostate-specific membrane antigen (PSMA) is a 100 kD, 750 amino acid (AA) long type II transmembrane glycoprotein that has a short N-terminal intracellular domain with 19 AA, 24 AA transmembrane proteins and a large C-terminal extracellular domain with 707 AA. PSMA has been mapped to chromosome 11p 11-12 in the region of the folate hydrolase gene (FOLH1) and has no known natural ligand. The protein possesses enzymatic activity—glutamate carboxypeptidase II (GCP-II)—and is thought to have role in folate uptake (FOLH1 gene). ‘PSMA’ expression, although significantly up-regulated in prostate carcinoma (more in high-risk and aggressive variants), is not exclusive for it and is noted in various other benign and malignant conditions, especially in the neovasculature. Currently, PSMA PET-CT is approved for high-risk and biochemically recurrent prostate carcinoma (PCa), and in patient selection for PSMA based theranostics. This review aims to highlight the clinical evolution of the PSMA molecule and PSMA PET-CT as a diagnostic modality, various indications of PSMA PET-CT, the appropriateness criteria for its use, pitfalls and artefacts, and other uses of PSMA PET apart from prostate carcinoma.

## 1. Brief Clinical History, Evolution and Various Types of PSMA-Targeting Agents

In 1987, Gerard Murphy, Julius Horoszewicz and colleagues developed 7E11-C5 (capromab), a murine monoclonal antibody against the human prostate cancer-derived cell line, LNCaP, and inferred that this new antigenic marker could be of potential clinical importance in prostate cancer [1]. Working with 7E11-C5 from 1993 to 1995, Warren Heston and William Fair of the Memorial Sloan Kettering Cancer Center (MSKCC) cloned the PSMA gene, described PSMA as folate hydrolase highly expressed in prostate cancer, and further detailed its tissue distribution and mapped its genomic organization on chromosome 11p11-12 [2,3,4,5,6,7].

In 1996, ^111^In-capromab (7E11-C5) pendetide (Prostascinct) became the first US FDA (United States Food and Drug Administration)-approved molecular imaging agent for prostate carcinoma. It targets only the intracellular domain of PSMA, binding only to dead/dying cells and in this way limiting the diagnostic performance, particularly in osseous metastases [8,9,10]. In 1997, Neil Bander and colleagues at Weill Cornell Medical College developed the first antibodies J591 to the extracellular domain of PSMA, and this enabled study of PSMA in viable cells [11]. Bander et al. also demonstrated that PSMA is constitutively internalised and that antibody binding significantly increases the rate of internalization [12]. Later, the humanised form of J591 (huJ591) was developed, offering increased clinical potential, and the antibody was radio-labelled with various alpha- (^225^Ac and ^213^Bi) and beta (^177^Lu and ^90^Y)-emitting agents and demonstrated promising anti-tumour activity [13,14,15,16].

In the twenty-first century (2001, specifically), Alan Kozikowski et al. at Georgetown University developed urea-based inhibitors of the central nervous system (CNS) neurotransmitter regulator GCP-II (NAALADase), regarded as the CNS version of PSMA [17]. The urea-based motif (glutamate-urea-lysine) binds with high affinity to the extracellular domain of PSMA (GCP-II) [18]. Martin Pomper of John Hopkins University recognised the potential of radiolabeling these PSMA-targeting agents for molecular imaging using positron emission tomography (PET) and single-photon emission computed tomography (SPECT) systems, and for radionuclide therapy as well [19,20]. Popper and colleagues further developed various PSMA-targeting PET agents, viz. ^18^F-DCFBC in 2008, ^18^F-DCFPyL (PyL) in 2011 and various ^68^Ga-labelled PSMA-targeted ligands [21,22,23]. The most commonly used PSMA PET tracer, ^68^Ga-PSMA-11 (also known as PSMA-HBED-CC or DKFZ-PSMA-11) was developed by Matthias Eder in 2012 at the German Cancer Research Center [24]. To date, many PSMA-targeting small molecular agents have been developed worldwide as derivatives of these early urea-based compounds, and have demonstrated high binding affinity, better internalization and rapid plasma clearance. Another important molecule of theranostic interest revolutionised the field of PSMA therapeutics; PSMA-617, which was developed and used by Eder, Clemens and Kratochwil in 2015 at University Hospital, Heidelberg, showed radiologic and PSA responses in metastatic castration-resistant prostate carcinoma (mCRPC) patients [25,26].

## 2. Structure, Normal Biodistribution and Function of PSMA

PSMA is a 100 kD, 750 amino acid (AA) long type II transmembrane glycoprotein that has a short N-terminal intracellular domain with 19 AA, 24 AA transmembrane protein and a large C-terminal extracellular domain with 707 AA [27,28]. PSMA has been mapped to chromosome 11p 11-12 in the region of folate hydrolase gene (FOLH1) and has no known natural ligand. The protein possesses enzymatic activity—glutamate carboxypeptidase II (GCP-II)—and is thought to have a role in folate uptake (FOLH1 gene) [6,29,30]. PSMA is normally expressed in all types of prostate tissue, but is over-expressed in the prostate cancer cells, proximal renal tubules, brush border of small intestines, salivary glands and some glial cells in the brain.

After binding to the extracellular domain, there is internalization of PSMA, and it binds to the intracellular domain having a novel MXXXL motif, which mediates internalization by reacting with the clathrin-adapter protein 2 complex [31]. The intracellular domain binds to the structural protein and other macromolecular complex that ultimately activate the protein kinase B (AKT) pathway and the mitogen-activated protein kinase pathway to promote proliferation and survival [32]. The enzymatic activity of PSMA is considered part of the co-catalytic zinc metallopeptidase family M28, and there is strong expression of PSMA in the proximal small intestine that could identify poly-*γ*-glutamated folate as a substrate [6,33]. Hence, PSMA/folate hydrolase 1 (FOLH1) has major role in folate uptake by removing *γ*-linked glutamates from folate, providing deglutamated folate for absorption and nutrition and leaving *α*-linked glutamate attached. In prostate and other carcinomas, PSMA plays a role in promoting carcinogenesis either by (a) providing folates for cell nutrition and survival, (b) decreasing cell-cycle time in G2/M by associating with anaphase-promoting complex culminating in aneuploidy and carcinogenesis, or through (c) *β*1 integrin-mediated activation of endothelial cells leading to enhanced angiogenesis [34,35,36,37]. The former two are implicated in prostate carcinogenesis, while the latter is seen in various other solid tumours and many benign conditions expressing PSMA. In benign prostate cells, PSMA is localised in the cytoplasm and the apical side of the prostate epithelium lining prostatic duct. However, post-malignant transformation, PSMA is transferred to the luminal side of the prostatic ducts, and in other non-prostatic solid tumours, PSMA is expressed in the tumour neovasculature.

## 3. Various Indications for PSMA-Targeted Imaging in Prostate Carcinoma

### Localisation of Intraprostatic Tumour

**Figure 1:**
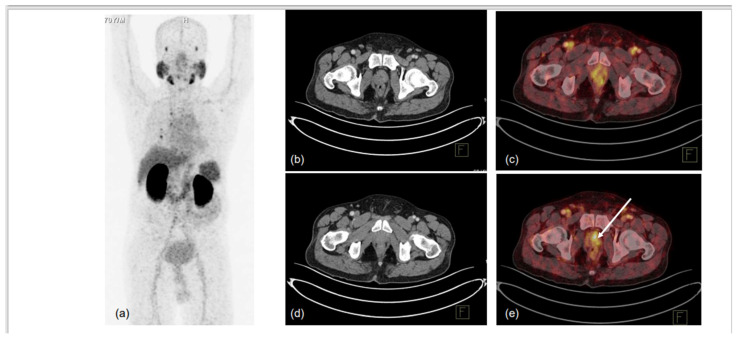
70-year-old male, presented with difficulty in urination and lower urinary tract symptoms (LUTS). Serum PSA was 0.43 ng/mL. mpMRI of prostate was performed, which showed indeterminate lesion in right peripheral zone of mid-gland region. ^68^Ga-PSMA-11 PET-CT was used to localise prostate lesion for targeted biopsy. MIP (**a**) showed low-grade PSMA uptake in prostate, with non-specific uptake in right lung and normal biodistribution. CT trans-axial slices and corresponding fused PET-CT trans-axial slices show mildly PSMA-avid hypodense lesion in right peripheral and central zones in mid-gland region (1.0 × 1.0 cm, SUVmax 5.6) (**b**–**e**). No locoregional or distant metastases were noted. TRUS-guided biopsy of PSMA-expressing lesion showed infiltrating acinar adenocarcinoma, Gleason grade 3 + 3/10. Arrow in ‘e’ denotes the PSMA-expressing area submitted for biopsy.

Localising the intra-prostatic tumour foci is one of the most important emerging indications of PSMA PET in the near future, in association with multi-parametric magnetic resonance imaging (mpMRI). PSMA PET in this setting will be helpful in detecting the tumour foci and subsequently guiding a targeted biopsy, particularly in patients with negative (Prostate Imaging-Reporting and Data System, PIRADS 1 or 2) or inconclusive (PIRADS 3) mpMRI findings and clinical or biochemical features highly suggestive of PCa, hence increases the diagnostic accuracy (Figure 1). Bodar et al. mapped foci of increased PSMA uptake within the prostate gland in 30 patients, studied prospectively with ^18^F-DCFPyL PET-CT before radical prostatectomy (RP) [38]. Subsequent targeted biopsy of these PSMA-avid lesions detected PCa in 28 of 30 patients (~93%), however, considering all the intra-prostatic lesions, the sensitivity and specificity for PSMA PET were 61.4% and 88.3% respectively. Chen et al. retrospectively studied mpMRI and PSMA PET, both alone and in a hybrid setting (PET-MRI), maintaining the final histopathology results as the standard of reference, and found improved detection of clinically significant PCa in 66 lesions in 54 patients before RP. The combined hybrid PET-MRI showed significantly better accuracy than mpMRI alone, especially in PIRADS 3 (inconclusive)-lesion sensitivity (89% vs. 76%) and specificity (96% vs. 88%) [39]. The ongoing prospective multicenter PRIMARY trial will measure and compare the sensitivity, specificity, positive predictive value (PPV) and negative predictive value (NPV) of both mpMRI and PSMA PET versus targeted biopsy [40]. These results will be used to determine the proportion of men who can safely avoid biopsy without compromising detection of clinically significant PCa.

## 4. Primary Staging

**Biopsy-naive with strong clinical suspicion of PCa:** PSMA PET-CT has high sensitivity, specificity and negative likelihood ratio for detecting prostate carcinoma in biopsy-naive patients with clinical and biochemical findings indicative of PCa and complementary mpMRI. In their systemic review and meta-analysis comprising 7 studies including 389 patients, Satapathy et al. found the pooled sensitivity, specificity, positive likelihood ratio and negative likelihood ratio for the initial diagnosis to be 97%, 66%, 2.86 and 0.05, respectively [41]. The negative likelihood ratio of 0.05 translates to a 20-fold decrease in the likelihood of PCa being present in patients with negative findings. Hence, PSMA PET has a high diagnostic accuracy for the initial detection of PCa that makes it a reliable “rule out” test in patients with clinical and biochemical findings indicative of PCa, thus ensuring that unnecessary prostate biopsies are safely avoided.

**Figure 2:**
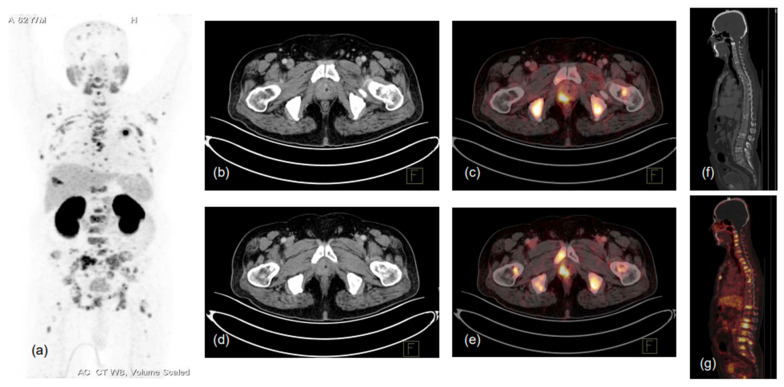
62-year-old male, presented with lower urinary tract symptoms (LUTS) and hematuria. USG of the whole abdomen showed prostatomegaly, and serum PSA was 480.43 ng/mL. TRUS-guided prostate biopsy showed infiltrating acinar adenocarcinoma, Gleason score 5 + 4 = 9. Patient was referred for PSMA PET-CT scan due to high-risk features. ^68^Ga-PSMA-11 PET-CT scan showed prostatomegaly with PSMA expressing heterogeneously, enhancing lesions in the right peripheral zone involving mid-gland and apical regions (SUVmax 17.0), with low-grade PSMA expressing a few tiny right internal iliac lymph nodes (SUVmax 2.8), and PSMA expressing multiple predominantly sclerotic and marrow lesions involving almost entire visualised skeleton (reference SUVmax 26.2 in sacrum). (**a**)-MIP image, (**b**–**g**)-CT and fused PET-CT trans-axial and sagittal images.

**Biopsy-proven PCa:** PSMA PET has shown promise for detecting nodal and distant metastasis of prostate carcinoma and is very useful for these indications in biochemically recurrent PCa (BCR). However, its performance in local staging-seminal vesicle invasion (SVI) and extra-prostatic extension (EPE) is not well established, although recent data appears quite promising, especially for PSMA PET-MRI in detecting SVI than EPE. A meta-analysis including over 9700 patients confirmed the sensitivity of MRI for SVI and EPE to be moderate and heterogeneous (57% and 58% for SVI and EPE, respectively). Hence, there is an evident clinical need to improve pre-operative risk assessment in patients with prostate cancer. In their systemic review and meta-analysis of 12 studies including 615 patients, Woo et al. found the pooled sensitivity and specificity of PSMA PET for detecting SVI to be 69% and 94%, respectively, and to be 72% and 87%, respectively, for detecting EPE [42]. Upon analyzing PET-CT versus PET-MRI, the sensitivity and specificity were found to be 60% vs. 87% and 96% vs. 91%, respectively, for SVI, and were 65% vs. 82% and 95% vs. 73%, respectively, for EPE [42]. Thus, PSMA PET (CT/MRI) is a “**one-stop shop**” imaging modality for primary staging of prostate carcinoma and helps in localizing the primary tumour, detecting locoregional and distant metastases for accurate assessment of SVI > EPE (PET-MRI > PET-CT) (Figure 2). In their prospective study, Kuten et al. compared the diagnostic accuracy of ^18^F-PSMA-1007 with that of ^68^Ga-PSMA-11 in 16 males newly diagnosed with intermediate- to high-risk PCa, and demonstrated that both tracers detected all prominent lesions in patients and ^18^F-PSMA-1007 detected additional low-grade lesions of limited clinical relevance [43].

## 5. Biochemical Recurrence and Metastatic PCa

**Figure 3:**
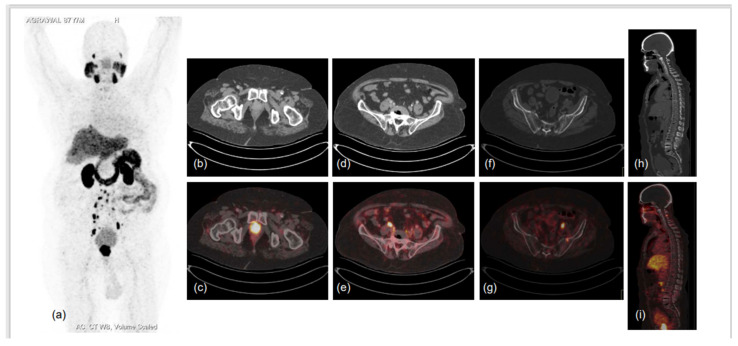
87-year-old male diagnosed with adenocarcinoma of prostate (Gleason score 7) in 2017 was heavily pre-treated: ADT with Pamorelin + Bicalutamide followed by Abiraterone and Enzalutamide. Nadir PSA was 0.016 ng/mL during the treatment and biochemical progression was observed for 6 months following. He presented to the doctor’s office with shortness of breath, limp legs, weakness and leg pain, his serum PSA was 94.7 ng/mL, and he was referred for PSMA PET CT scan. ^68^Ga-PSMA-11 PET CT scan showed prostatomegaly invading the urinary bladder wall with PSMA expressing irregular enhancing SOL involving almost entire gland. PSMA expressing multiple metastatic pelvic and retroperitoneal lymph nodes and PSMA expressing multiple sclerotic metastatic skeletal lesions showed disease progression as compared to previous PSMA PET-CT scan of 2019. (**a**)-MIP image, (**b**–**i**)-CT and fused PET-CT trans-axial and sagittal images.

Biochemical recurrence is defined by a post-RP PSA level of >0.2 ng/mL that rises on at least two consecutive measures taken at least 3 weeks apart and post radical EBRT, such as a rise of 2.0 ng/mL or more above the nadir value that occurs more than 6 weeks after RT conclusion. Biochemical recurrence after radical prostatectomy and radiotherapy occurs in up to half of patients with PCa, and more than a quarter of these patients eventually experience clinical recurrence in approximately 7 to 8 years [44]. The diagnostic yield of conventional imaging modalities, such as bone scans and CT scans, ranges from 5% to 14% in patients with PSA values less than 7 ng/mL [45]. PSMA PET has a better detection rate and diagnostic accuracy than conventional imaging for BCR, because of its remarkable ability to detect metastasis/es in low-volume disease and low serum PSA levels (Figure 3).

Various studies have reported detection rates ranging from 75% to 90% [45,46,47]. Fendler and colleagues have demonstrated positive predictive values of 84% to 92% with a 75% overall detection rate in patients with BCR and median PSA of 2.1 ng/mL [47]. In another study, Afshar-Oromieh et al. detected PCa in 83% (264 of 319) patients with BCR, and with high specificity [45]. Eiber et al. reported diagnostic accuracy of 89.5% (222 of 248 patients) and showed a positive correlation between PSA level-detection rates of 96.8%, 93%, 72.7% and 57.9% and PSA values of >/=2.1 ng/mL, <2.0 ng/mL to 1.0 ng/mL, <1.0 ng/mL to 0.5 ng/mL and <0.5 ng/mL to 0.2 ng/mL, respectively [46]. In their meta-analysis that included 16 articles and 1309 patients, Perera and colleagues reported a 76% detection rate for BCR that varied with PSA values, as 0 to 0.2 ng/mL was 42%, 0.2 to 1 ng/mL was 58%, 1 to 2 ng/mL was 76% and >2 ng/mL was 95% [48]. On a per-patient basis, the sensitivity and specificity were both 86%, and on a per-lesion basis, the sensitivity and specificity were 80% and 97%, respectively [48].

Among the different modalities, PSMA PET detected sites of recurrence when the PSA level was as low as <1.0 ng/mL; by comparison, bone scans detected osseous metastases at a median PSA value of 40 ng/mL [49]. Abuzallouf et al. showed osseous detection rates of bone scans to be 2.3% for PSA < 10 ng/mL, 5.3% for PSA 10.1 to 19.9 ng/mL and 16.4% for PSA 20 to 49.9 ng/mL, and lymph node detection rates of CT scans to be 0% for PSA < 20 ng/mL and 1.1% for PSA > 20 ng/mL [50]. In contrast, PSMA PET identified 51.5% patients as having potential sites of recurrence detected at PSA < 1.0 ng/mL, which increased to 74% at PSA > 1.0 ng/mL and surpassed 90% when PSA was >2.0 ng/mL.

Metastatic PCa can be hormone-sensitive and castration-resistant where the PCa cells are refractory to androgen deprivation therapy and there is disease progression even at castrate levels of testosterone, and PSMA uptake (PSMA expression) is decreased in mCRPC as compared to hormone sensitive disease [51]. Apart from oligometastatic disease, in metastatic PCa, precise disease localization is less important than mapping disease extent. PSMA PET could be highly impactful in disease mapping in mCRPC settings and was found to detect unsuspected metastatic lesions in 55% of patients who were labeled as non-metastatic by conventional imaging [52].

## 6. Treatment Planning and Theranostic Application

**Figure 4:**
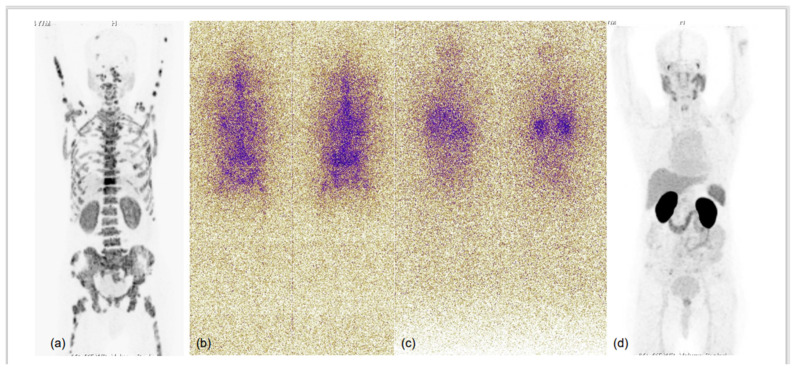
75-year-old male with known case of coronary artery disease, post-CABG and on cardiac remodeling agents, presented with multiple sites of skeletal pain, weakness and high serum PSA >500.0 ng/mL. Needle biopsy from prostate demonstrated acinar adenocarcinoma (Gleason score 4 + 3 = 7), underwent bilateral orchidectomy. ^68^Ga-PSMA-11 PET-CT performed for high-serum PSA showed low-grade PSMA expression in relatively smaller prostate gland, with no significant PSMA-expressing or otherwise pelvic and retroperitoneal lymph nodes and PSMA-expressing sclerotic and marrow metastatic lesions involving entire visualised skeleton. In view of cardiac co-morbidity and post-CABG status, chemotherapy and anti-androgen therapies were not considered, and he was taken for ^225^Ac-PSMA-617 (alpha radionuclide) therapy. MIP images of ^68^Ga-PSMA-11 PET-CT scans at baseline (**a**) and 3 months after second cycle of ^225^Ac-PSMA-617 (**d**), and first (**b**) and second (**c**) post alpha therapy planar gamma scans showed excellent scan response; PSA decreased to 0.405 ng/mL and patient became asymptomatic.

PSMA PET is complementary to mpMRI in initial staging of PCa and can accurately determine which patients will benefit from definitive RP or radical RT and which are more likely to have biochemical failure and occult clinical relapse depending on SVI, EPE and whether they are not candidates for definitive therapy, such as for those with distant lymph nodal, visceral and osseous involvement. Further PSMA PET, along with mpMRI or hybrid PET-MRI, can accurately select the patients without EPE who can safely undergo nerve-sparing surgery, mainly with the aim of reducing post-operative urinary incontinence and erectile dysfunction [53]. In metastatic PCa, PSMA PET is of crucial importance for treatment planning by effectively diagnosing oligometastatic PCa, determining who can be amenable to metastasis-directed therapy (MDT): cases of metastatic disease with either nodal, osseous or visceral involvement for which chemotherapy and newer anti-androgens are the therapeutic modalities of choice, or bone-predominant diseases which are better treated by ^223^Radium (^223^Ra) or other alpha emitters. Last but not least, avid PSMA uptake (expression) in mCRPC lesions can be effectively targeted by radio-ligand therapy (RLT) using ^177^Lutetium (^177^Lu), ^225^Actinium (^225^Ac) or ^213^Bismuth (^213^Bi) [54,55,56,57,58,59,60,61,62,63,64] (Figure 4).

**Figure 5:**
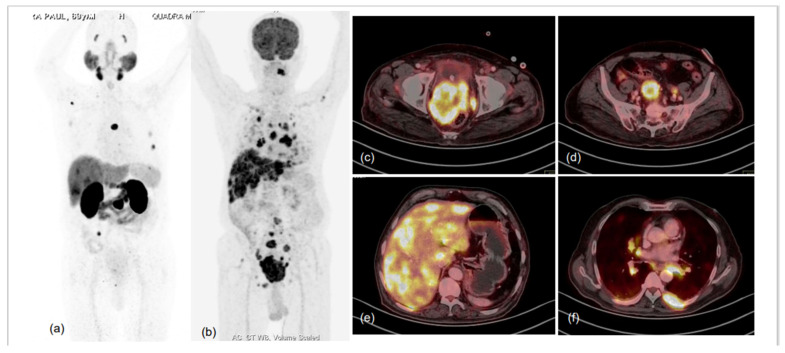
76-year-old male, attended urologist’s office for lower urinary tract symptoms (LUTS) and prostatomegaly; suspected prostate carcinoma, underwent biochemical evaluation, and serum PSA was 1.120 ng/mL. TRUS-guided prostate biopsy was performed and was diagnosed as high-grade acinar adenocarcinoma (Gleason score 5 + 5 = 10); IHC tumour cells were found to express cytokeratin, EMA, TTF1, synaptophysin, chromogranin A and Mib-1-labelling index of approx. 75%. Hence, a diagnosis of high-grade acinar adenocarcinoma of prostate with small-cell transformation was made and the patient was taken for dual-tracer PET with ^68^Ga-PSMA-11 and FDG after deliberate discussion with referring urologist and taking him and the patient into confidence. MIP images of ^68^Ga-PSMA-11 (**a**) and FDG (**b**) PET CT scans demonstrated significant discordance in terms of number and intensity of tracer uptake in the lesions, with significantly more lesions showing FDG uptake of increased intensity. Fused trans-axial PET and CT slices of FDG PET-CT (**c**–**f**) showed FDG-avid lesions with high-grade metabolic activity with visceral (hepatic) and lytic skeletal lesions. The results were in accordance with the known phenomenon of small-cell prostate carcinomas being more aggressive, showing lesser PSMA expression in lesions and being metabolically active due to rapid proliferation.

**Figure 6:**
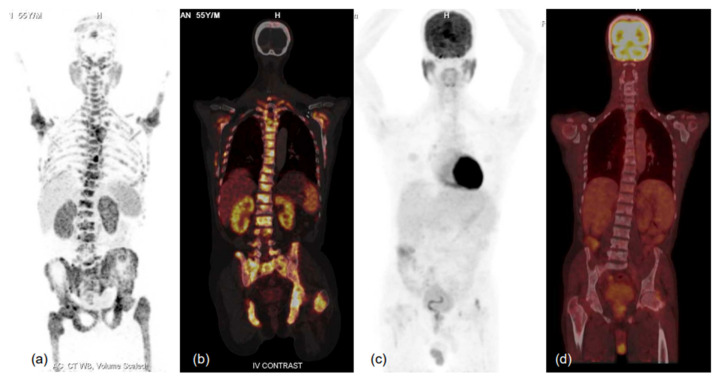
MIP images of ^68^Ga-PSMA-11 (**a**,**b**) and FDG (**c**,**d**) PET-CT scans and corresponding fused coronal images (b-^68^Ga-PSMA-11 and d-FDG PET-CT scans) in bone window showing PSMA-expressing multiple sclerotic and marrow lesions involving almost the entire axial and proximal appendicular skeleton, while no significant FDG uptake (metabolic activity) is evident in the lesions. Such patients respond well to therapy, especially PSMA-targeting radionuclide therapy, and demonstrate relatively favourable prognoses in terms of survival and quality of life benefits. Thus, dual-tracer PET using FDG and ^68^Ga-PSMA-11 helps in patient selection for PSMA peptide receptor radionuclide therapy (PRLT) and in predicting treatment outcomes. He was scheduled for ^177^Lu-PSMA-617 therapy, but unfortunately could not attend due to logistical constraints.

Low-grade to insignificant PSMA uptake (expression) in metastatic prostate carcinoma can point towards the development of treatment-emergent small cell neuroendocrine carcinoma of prostate, a poorly understood and highly aggressive variant of PCa. Small-cell neuroendocrine PCa often shows avid FDG uptake, suggesting a possible role of FDG-PET in PCa and, hence, of dual-tracer imaging (Figure 5). A recent review studying the role of dual-tracer PET-CT (using PSMA and FDG) for precision radio-molecular theranostics in PCa highlighted the utility of this concept in providing a better understanding of tumour biology in various clinical settings [65], and another retrospective study from the same authors had proposed an integrated scoring system for mCRPC using PSMA and FDG PET (Pro-PET score) for prognostication and therapeutic selection through the ‘Pro-PET’ scoring system [65,66] (Figure 6). In another study by Alberts et al., the role of dual-tracer PET-CT using FDG and PSMA single-day protocol was studied using a long-axial field-of-view scanner. The authors found that the dual-tracer PET approach was able to reveal lesions with low PSMA-avidity which resulted in higher sensitivity as compared to ^68^Ga-PSMA-11 PET-CT alone [67].

## 7. Treatment Response Evaluation and Modulation of PSMA Expression by ADT

The unique ability of PSMA PET to image the tumour directly, unlike many conventional imaging techniques in use, establishes its potential for use in treatment response evaluation, and early findings have been promising. Another unique and remarkable ability of PSMA PET is to detect lesions in prostate and soft tissue sites including lymph nodes while also detecting visceral involvement and osseous metastases, all in a single imaging study with high sensitivity and specificity. Emerging data show PSMA PET to be useful in treatment response evaluation after definitive therapy-RP and -RT, hormonal therapy, taxane-based chemotherapy and PSMA-targeting RLT. Some caution, however, is warranted in view of ‘flare phenomenon’ due to transiently increased PSMA expression after hormonal therapy and in treatment-emergent small-cell neuroendocrine carcinoma of prostate, which shows low-grade to near-zero uptake, and FDG-PET can be complementary in such cases. Hence, it is of pivotal importance to define a comprehensive response criterion for PSMA PET, and further research is needed.

An enigmatic yet intriguing phenomenon of dichotomous PSMA expression in response to androgen-deprivation treatment is variable PSMA expression in hormone-sensitive (decreased) and castration-resistant (increased) metastatic prostate carcinoma. On one hand, this can potentially limit the treatment response evaluation using PSMA PET, but on the other hand, can prognosticate by predicting lesions which are at higher risk of becoming castration-resistant in the future. The possible genetic mechanism of PSMA expression is through regulation via the FOLH1 gene with the help of two regulatory elements—the PSMA promoter and PSMA enhancer—located within the third intron of the FOLH1 gene in castration-resistant PCa [68]. FOLH1 gene expression is downregulated by androgens that reduce the transcription of PSMA mRNA. Hence, anti-androgen up-regulates the FOLH1 gene expression, leading to increased PSMA uptake in castration-resistant PCa. However, in vivo studies have showed decreases in tumour size in response to ADT; in summary, ADT administration may lead to increased PSMA uptake on PSMA PET imaging due to androgen receptor inhibition, but that androgen receptor inhibition long-term causes PCa cell death and reduction in tumour mass [69]. Afshar-Oromieh et al. studied the effect of long-term (mean 7 months) ADT in 10 hormone-sensitive PCa and found that PSMA uptake decreased in approximately 75% of lesions, whereas in a small proportion (13%) of lesions, PSMA uptake increased despite a complete or partial PSMA response [70]. The authors postulated that the lesions which showed an increased PSMA uptake despite clinical and PSA response might reflect those cell clones that will become castration-resistant first.

## 8. Radio-Metal Based PSMA Tracers Versus Prosthetic Group-Based PSMA Tracers

Radio-metal based PSMA-targeting PET tracers using bifunctional chelating agents (BFCA) are the most commonly used PSMA-targeting PET radio-tracers, and are FDA-approved for imaging prostate carcinomas. Most of the available data on and experiences of PET imaging in prostate carcinomas targeting PSMA are with ^68^Gallium (68Ga)-labeled urea-based small molecules such as PSMA-HBED-CC (or PSMA-11) and PSMA-617, of which the latter has been tagged with ^177^Lu/^225^Ac for therapeutic applications. The advantages of using radio-metal agents using BFCA are in-house elution and synthesis, no need for a technically demanding and costly cyclotron setup, a fairly good diagnostic potential and reliable theranostic pair. The major disadvantages include the relatively high background noise due to high energy positrons, higher urinary excretion, relatively higher radiation dose to patients and the need to frequently replace the generator as well as the lower yield at the end of the generator’s life. Prosthetic group-based PET tracers using ^18^Fluorine (^18^F) are recently becoming popular due to a longer half-life that permits the tracer to be transported from production sites; this allows for delayed imaging, lesser noise and more photons, leading to smoother images and negligible urinary excretions, making detection of pelvic lesions easier and more accurate (PSMA-1007, on the other hand, shows hepatobiliary excretion). Recent studies have shown better detection rates with ^18^F-PSMA-1007 PET-CT, particularly at low PSA levels, in suspected biochemical recurrence after radical prostatectomy and/or definitive RT. The major drawback with ^18^F based agents is the non-availability of theranostic pairs for PRLT. Table 1 provides a head-to-head comparison between the two classes of tracers.

## 9. Appropriateness Use Criteria (Auc) of PSMA Based PET-CT

Published in March 2022, by Jadvar et al., appropriateness use criteria for PSMA PET imaging refers to a score of 1 to 9 that correlates to a set of clinical scenarios where use of PSMA PET, considering current clinical evidence and mutual consensus, categorises them as: (a) appropriate—7 to 9, (b) may be appropriate—4 to 6 and (c) rarely appropriate—1 to 3 [71].

Various appropriateness use scenarios in decreasing order of appropriateness with the individual scores in the parentheses are described below:

1. **Appropriate:** PSA persistence or PSA rise from undetectable level after RP—9; PSA rise above Nadir after definitive radiotherapy—9; evaluation of eligibility for PSMA-targeted PRLT—9; newly diagnosed unfavourable intermediate, high risk or very high risk PCa—8; newly diagnosed unfavourable intermediate, high risk or very high risk PCa with negative/equivocal or oligometastatic disease on conventional imaging—8; non-metastatic CRPC (nmCRPC, M0) on conventional imaging—7.
2. **May be appropriate:** PSA rise after focal therapy of the primary tumour—5, post-treatment PSA rise in the mCRPC setting for a patient not being considered for PSMA-targeted RLT—5, evaluation of response to therapy—5, newly diagnosed PCa with widespread metastatic disease on conventional imaging—4.
3. **Rarely appropriate:** Patients with suspected PCa (eg. high/rising PSA levels, abnormal digital rectal examination results) evaluated for targeted biopsy and detection of intraprostatic tumour—3; patients with very low, low and favourable intermediate risk PCa—2.

## 10. Artefacts and Pitfalls

### 10.1. Common Artefacts Encountered in PSMA PET-CT Are

Halo artefacts: Caused due to high adjacent activity (e.g., high activity in kidneys, urinary bladder and associated structures) and may impede visualization of regional lymph nodes and osseous uptake. This can be improved by delayed imaging of the pelvis after bladder voiding and administration of diuretic agents.

Motion artefacts: Caused by patient and respiratory motion and lead to mis-registration between anatomical structures and PSMA uptake. This is more pronounced in the areas in proximity to diaphragm as well as the patient’s extremities.

Flare phenomenon: Happens following initiation of ADT with a gonadotropin-releasing hormone (GnRH) antagonist, where lesions may show an increased standardised uptake value (SUV) by up to 73% after 2 weeks. Additional lesions may also be visible following flare phenomenon, and the appropriate time for PSMA PET for lesion detection is 2 to 4 weeks after initiation of ADT.

### 10.2. Common Imaging Pitfalls in PSMA PET-CT Are

PSMA PET is a highly sensitive and specific imaging modality, but a variety of pathophysiological processes can express PSMA and result in interpretative error. Functionally, PSMA is folate hydrolase, which is expressed in a variety of normal tissues, tissue neovasculature and other tumour types, both benign and malignant. Normal physiological PSMA uptake is demonstrated in the lacrimal gland, parotid and submandibular salivary glands, liver, spleen, bowel (specially duodenum > other small bowel) and urinary tract. Low-grade PSMA uptake is seen in the larynx, oesophagus and stomach due to salivary excretion, and in the gall bladder and biliary ducts due to hepatobiliary clearance. Low-grade physiological activity is also appreciated in sympathetic ganglia (celiac, stellate, hypogastric and pre-sacral) and trigeminal ganglia in Meckel’s cave.

Infection and inflammation: Currently, little is known regarding immune cell PSMA expression and possible mechanisms include neovascularization, macrophage folate receptors, increased vascular flow and permeability [72]. Non-prostatic infective/inflammatory processes include neurocysticercosis, tuberculosis, diverticulosis, post-surgical inflammatory changes, etc. Inflammatory prostate uptake may also be seen in the prostate bed and prostatic urethra following RP and RT and are usually reported to persist for approximately 2 months. Infective and inflammatory pulmonary nodules demonstrate low-grade focal uptake.

Bone conditions: Malignant involvement of bones and bone marrow on PSMA PET shows high-grade to intense focal uptake. Low-grade or more diffused uptake patterns have been observed in benign conditions, such as fracture, osteomyelitis, Paget’s disease, fibrous dysplasia, hemangiomas and osteophytes.

Benign neoplasms: PSMA uptake in benign neoplasms is supposed to involve soft tissue and abnormal vascular proliferation. PSMA uptake has been also noted in meningiomas, nerve sheath tumours, schwannomas and other neurogenic tumours. Soft tissue lesions demonstrated to show PSMA uptake include thyroid and parathyroid adenomas, adrenal adenomas, thymomas and dermatofibromas. Low-grade PSMA expression is also noted in gynecomastia and soft tissue hemangiomas.

Non-prostate malignant neoplasms: High-intensity uptake is reported in renal cell carcinoma, glioblastoma multiforme, hepatocellular carcinoma, salivary gland ductal carcinoma and pulmonary adenocarcinoma, and are mainly attributed to tumour neovasculature than to tumour cells [73,74,75]. Low-intensity PSMA uptake is reported in breast carcinoma, lymphoma, meningioma, squamous cell carcinoma, and well-differentiated thyroid carcinoma (particularly iodine refractory types) [76,77,78].

In a recently published study, Vollnberg and colleagues demonstrated that uncertain focal bone uptake (UBU), frequently encountered on ^18^F-PSMA-1007 PET-CT, can pose a diagnostic conundrum and may lead to incorrect staging, particularly after the advent and increased availability of ultrasensitive digital and long-field-of-view PET-CT systems. They found that 1/11 (9.1%) of the bone foci biopsied was confirmed as metastatic of prostate carcinoma while 10/11 (90.9%) foci were found to be unremarkable. They inferred that UBU on ^18^F-PSMA-1007 must be interpreted with caution, so as to minimise the risk of erroneous over-staging and subsequent treatment [79].

## 11. Uses of PSMA PET Other Than Prostate Carcinoma

In view of the aforementioned findings, various non-prostatic malignancies showing intense PSMA uptake, viz. renal cell carcinoma, glioblastoma multiforme, hepatocellular carcinoma, salivary gland ductal carcinoma and pulmonary adenocarcinoma can be imaged using PSMA-based PET, and further research is required to establish the sensitivity, specificity, PPV, NPV and diagnostic accuracy for these indications. Furthermore, high-grade PSMA expression (evident by avid PSMA uptake) can be helpful in patient selection for theranostic interventions. Gundogan and colleagues compared PSMA PET and FDG PET for imaging hepatocellular carcinoma (HCC) in 14 patients and demonstrated PSMA-PET to be superior to FDG PET in staging of HCC [80].

## 12. Conclusions

PSMA-targeting ligands for PET imaging in prostate carcinoma has been embraced at an unprecedented rate and has been incorporated into the diagnostic flowchart of PCa. Several small-molecule PSMA PET radiotracers are now available, increasing the availability of PSMA PET worldwide. There is a need for nuclear medicine physicians to familiarise themselves with a standardised reporting system, in order to have a strict collaboration with the clinicians for effectively interpreting and implementing the imaging findings for clinical and academic benefits. Although PSMA PET is currently FDA approved in PCa for imaging biochemical recurrence and high-risk cases, its use in other clinical scenarios of PCa and for few other malignant conditions has been encouraging. Furthermore, a sound knowledge of physiological biodistribution and uptake in various benign and malignant conditions is of key importance to avoid imaging pitfalls and artefacts. 

## Figures and Tables

**Table 1:** Head-to-head comparison of 18F (prosthetic-based) and BFCA chelated radio-metal-based tracers targeting PSMA for imaging prostate carcinoma.

Characteristics	^18^F (Prosthetic-Based) Tracers	Radio-Metal-Based Tracers Using BFCA
**Physical, Chemical and Radio-biological aspects**		
Source	Cyclotron	^68^Ge/^68^Ga Generators
Radiochemistry	Relatively lower energy positron causing less image noise. Higher positron yield.	High energy positron causing increased image noise. Low positron yield.
Radiation Safety	Relatively less radiation exposure	Higher radiation exposure
Synthesis	Time consuming and challenging	Relatively simpler and less time consuming
Yield	Remains stable	Decreases as the generator gets old
Installation and maintenance	More technically demanding and costly	Less technically demanding and relatively less costly
**Pharmacokinetic properties**		
Half-life (t1/2)	Longer half-life: permits transportation, longer uptake time and delayed imaging	Shorter half-life mandates an in-house generator and cannot be transported
Binding affinity	Relatively lesser	Strong binding affinity
Excretion	Hepatobiliary (PSMA 1007) and Urinary (DCFPyL) PSMA 1007 is better for imaging pelvic disease	Predominately urinary smaller pelvic lesions may remain obscured due to high urinary activity
**Detections Rates**		
Overall for putative sites of disease on a per-patient-based analysis	Higher detection efficiency (DCFPyL > 1007)	Relatively lesser than ^18^F-based agents
According to PSA levels	Demonstrates better statistics at lower level of PSA	Demonstrates relatively poor statistics at lower PSA but has similar detection rates at PSA > 1.0 to 2.0 ng/ml
In accordance with histopathology and Gleason score	No significant correlation with Gleason score/grade	Correlates well with Gleason score/grade
**Theranostic potential**		
^177^Lu/^225^Ac-based agents	No known theranostic pair in widespread clinical use. ^177^Lu-CTT 1403 is theranostic pair of ^18^F-CTT 1057 and is in pre-clinical trials	^177^Lu-PSMA-617 is a good theranostic pair which has widespread clinical use with good results and has recently received FDA approval
Tracers	PSMA-1007, PSMA-DCFBC, PSMA-DCFPyL	PSMA-HBED-CC (PSMA-11), PSMA-617, PSMA- I and T.
